# Activation volume in superpressed glass-formers

**DOI:** 10.1038/s41598-019-49848-w

**Published:** 2019-09-24

**Authors:** Aleksandra Drozd-Rzoska

**Affiliations:** Institute of High Pressure Physics Polish Academy of Sciences, ul. Sokołowska, 29/37.01-142 Warsaw, Poland

**Keywords:** Condensed-matter physics, Glasses, Liquid crystals, Polymers

## Abstract

In pressurized glass-forming systems, the apparent (changeable) activation volume *V*_*a*_(*P*) is the key property governing the previtreous behavior of the structural relaxation time (*τ*) or viscosity (*η*), following the Super-Barus behavior: ${\boldsymbol{\tau }}{\boldsymbol{(}}{\boldsymbol{P}}{\boldsymbol{)}}{\boldsymbol{,}}{\boldsymbol{\eta }}{\boldsymbol{(}}{\boldsymbol{P}}{\boldsymbol{)}}{\boldsymbol{\propto }}{\bf{\exp }}{\boldsymbol{(}}{{\boldsymbol{V}}}_{{\boldsymbol{a}}}{\boldsymbol{(}}{\boldsymbol{P}}{\boldsymbol{)}}{\boldsymbol{/}}{\boldsymbol{R}}{\boldsymbol{T}}{\boldsymbol{)}}$, *T* = *const*. It is usually assumed that *V*_*a*_(*P*) = *V*^#^(*P*), where ${{\boldsymbol{V}}}^{{\boldsymbol{\#}}}{\boldsymbol{(}}{\boldsymbol{P}}{\boldsymbol{)}}={\boldsymbol{R}}{\boldsymbol{T}}{\boldsymbol{d}}\,{\boldsymbol{ln}}\,{\boldsymbol{\tau }}{\boldsymbol{(}}{\boldsymbol{P}}{\boldsymbol{)}}{\boldsymbol{/}}{\boldsymbol{d}}{\boldsymbol{P}}$ or ${{\boldsymbol{V}}}^{{\boldsymbol{\#}}}{\boldsymbol{(}}{\boldsymbol{P}}{\boldsymbol{)}}{\boldsymbol{=}}{\boldsymbol{R}}{\boldsymbol{T}}{\boldsymbol{d}}\,{\boldsymbol{ln}}\,{\boldsymbol{\eta }}{\boldsymbol{(}}{\boldsymbol{P}}{\boldsymbol{)}}{\boldsymbol{/}}{\boldsymbol{d}}{\boldsymbol{P}}$. This report shows that *V*_*a*_(*P*) ≪ *V*^#^(*P*) for *P* → *P*_*g*_, where *P*_*g*_ denotes the glass pressure, and the magnitude *V*^#^(*P*) is coupled to the pressure steepness index (the apparent fragility). *V*^#^(*P*) and *V*_*a*_(*P*) coincides only for the basic Barus dynamics, where *V*_*a*_(*P*) = *V*_*a*_ = *const* in the given pressure domain, or for *P* → 0. The simple and non-biased way of determining *V*_*a*_(*P*) and the relation for its parameterization are proposed. The derived relation resembles Murnaghan - O’Connel equation, applied in deep Earth studies. It also offers a possibility of estimating the pressure and volume at the absolute stability limit. The application of the methodology is shown for diisobutyl phthalate (DIIP, low-molecular-weight liquid), isooctyloxycyanobiphenyl (8*OCB, liquid crystal) and bisphenol A/epichlorohydrin (EPON 828, epoxy resin), respectively.

## Introduction

Previtreous changes of the structural (primary, alpha) relaxation time (*τ*), viscosity (*η*), electric conductivity (*σ*), heat conductivity (*κ*), diffusion (*d*) or chemical reactions rates (*k*) in systems ranging from low-molecular-weight liquids and polymers to liquid crystals and plastic crystals are the key manifestation of the hypothetical universal dynamics emerging on approaching the glass transition (*T*_*g*_, *P*_*g*_)^1–5^. Similar patterns are observed both for the temperature and pressure paths^6–8^. The temperature path is associated with the Super-Arrhenius (SA) dynamics, and it is governed by changes of the apparent activation energy *E*_*a*_(*T*), which strongly increases on approaching the glass transition temperature *T*_*g*_^9–11^. The non-biased way of determining *E*_*a*_(*T*) and its properties are discussed in refs^12–14^ and recalled in Supplementary Info.

This report focuses on the still puzzling case of the (high) pressure-induced glass transition. For compressed glass-formers, general features of the previtreous dynamics are described by the Super-Barus (SB) equation^7–10^:

1 $$\tau (P)={\tau }_{0}^{P}\,{\exp }(\frac{P{V}_{a}(P)}{RT}),\,\,\eta (P)={\eta }_{0}^{P}\,{\exp }(\frac{P{V}_{a}(P)}{RT})$$

where *T* = *const* and *P* < *P*_*g*_; *V*_*a*_(*P*) denotes the apparent activation volume, which changes on compressing. Generally, the name ‘activation volume’ is reserved for the basic Barus^15^ equation with *V*_*a*_(*P*) = *V*_*a*_ = *const* in the given domain of pressures.

Prefactors *τ*_0_^*P*^ and *η*_0_^*P*^ in Eq. (1) refer to *P* = 0, but within the experimental error they can be approximated by atmospheric pressure values, i.e., *τ*_0_^*P*^ = *τ*(*P* = 0) ≈ *τ*(*P* = 0.1 *MPa*) for the tested isotherm *T*. For high-pressure studies the experimental errors are Δ*P* ≈ ±0.2 *MPa* (moderate pressures) and Δ*P* > ±1 *MPa* (GPa domain). The shift of pressure by 0.1 MPa does not yield detectable changes of dielectric relaxation time^6–8^.

Similar SB dependences describe pressure changes of all physical properties recalled above: pressure dependences of *τ*(*P*) and *η*(*P*) are parallel (Eq. (1))^6–8^, but for the remaining dynamic properties the translational - orientational decoupling have to be taken into account^8,10^. For instance, for DC electric conductivity^16^:

2 $${\sigma }^{-1}(P)={\sigma }_{0}^{-1}(S\frac{P{V}_{a}(P)}{RT})$$

where *S* < 1 is the decoupling exponent associated with the fractional Debye-Stokes-Einstein (f-DSE) dependence *σ*(*P*)[*τ*(*P*)]^*S*^ = *C* *=* *const*^8,10,16^.

The first discussion regarding *η*(*P*) or *τ*(*P*) behavior in compressed liquids can be associated with the relation $\eta (P)\propto {\exp }(\alpha P)$ proposed by Barus at the end of the 19^th^ century when studying viscosity of natural oils^15^. Whalley^17,18^ and Williams^19^ applied such description for pressurized polymers and dielectric relaxation time, introducing the activation volume *V*_*a*_, what led to Eq. (1) with *V*_*a*_ = *const*. The Barus (B) or Barus-Williams dependence can be considered as the pressure counterpart of the basic, temperature-related Arrhenius equation which was originally introduced as $k(T)={k}_{0}{\exp }({E}_{a}/RT)$, where *E*_*a*_ stands for the activation energy and *k* is the reaction rate coefficient^20^. Generally, for ultraviscous/ultraslow glass-forming systems one should expect the Super-Barus (SB) behavior (Eqs (1) and (2)), where dynamic properties are governed by changes of the pressure-dependent activation volume: the apparent activation volume.

According to the above discussion one concludes that *V*_*a*_(*P*) governs the dynamics of ultraviscous/ultraslow systems, and its determination and understanding is the key to the ultimate insight into the glass transition problem^6–9^, the behavior of soft matter under pressure^7–9,21^, high-pressure chemistry and biochemistry^22^, innovative material engineering^23^, high-pressure biotechnology^24^, geophysics, and deep Earth science^25^.

Usually, for the previtreous domain the apparent activation volume *V*_*a*_(*P*) is calculated from *τ*(*P*) or *η*(*P*) experimental data via^26–55^:

3 $${V}_{a}(P)\to {V}^{\#}(P)=RT[\frac{d\,{ln}\,\tau (P)}{dP}]$$

under the assumption that *V*_*a*_(*P*) = *V*^#^(*P*) and for 0.1 *MPa* < *P* ≤ *P*_*g*_.

The analysis exploring the (apparent) activation volume determined via Eq. (3) is the key point of numerous research reports. In the framework of the transition state theory, the activation volume describes the difference between volumes occupied by a molecule in activated and non-activated states^22^. It is the essential parameter characterizing the sensitivity of the structural relaxation time or other dynamic properties to pressure changes^7,8,21^. It estimates the local volume required for a given dynamical process (in the case of *τ* denotes molecular rearrangements)^8,26–35^. Hong *et al*.^36,37^ indicated that the activation volume correlates with the length scale of dynamical heterogeneities *ξ*^3^, which are considered as one of the essential sources of the previtreous ‘universality’ of dynamic previtreous properties. Tests in hydrogen-bonded molecular liquids showed the case-sensitivity of the activation volume, determined via Eq. (3) to subtle features of molecular structures^38–40^. Worth stressing is also the broadly used link between the activation volume at the glass transition, and the fragility: Δ*V*^#^ = *m*_*P*_ × 2,303*R*(*dT*_*g*_/*dP*)^8,40–42^, where the fragility ${m}_{P}={[d{lo}{{g}}_{10}\tau (T)/d({T}_{g}/T)]}_{T={T}_{g}}$ is one of key ‘universality’ metrics for the glass transition phenomenon^2–5,9,10,43,44^. The analysis via Eq. (3) was also used for showing different activation volumes determined by dielectric and light scattering spectroscopies^8,45–53^. Reasonings based on such analysis can yield important checkpoints for glass transition models^8,45–55^. The activation volume is also significant for the thermodynamic scaling linking *τ*(*T*.*P*, *V*) experimental data^8,54,55^. There are also reports where *V*^#^(*P*) is recalled as the (apparent) activation volume, but the link to the steepness index *m*_*T*_(*P*) is indicated^56,57^.

For the validation of Eq. (3) reports by Whalley^17,18^ and Williams^19^ are most often cited^8,30–34,38–42,48–54^. However, these reports did not consider the Super-Barus dynamics with the pressure depending apparent activation volume but the basic Barus behavior with the constant activation volume. This issue is worth stressing, since the SB Eq. (1) directly yields the differential equation:

4 $$RT\frac{d\,{ln}\,\tau (P)}{dP}={V}_{a}(P)+P\frac{d{V}_{a}(P)}{dP}$$

Comparing Eqs (3) and (4) one obtains that generally: *V*_*a*_(*P*) ≠ *V*^#^(*P*). The second term in Eq. (4) disappears only for two ‘special’ cases: (**i**) for *P* = 0, or (**ii**) for *V*_*a*_(*P*) = *V*_*a*_ = *const*, i.e., for the basic Barus behavior in the given pressure domain.

Worth recalling is the difference between the free volume (*V*_*f*_) as the volume not occupied by molecules and the activation volume (*V*_*a*_) as the volume required for the given process, for instance, the molecular rearrangement or reorientation. Then, one can expect *V*_*f*_  > *V*_*a*_^8,9^.

Consequently, the question arises for the (proper) estimation of the (apparent) activation volume in the previtreous domain. This report proposes the solution to this problem and discusses the meaning and behavior of both *V*^#^(*P*) and *V*_*a*_(*P*) for *P* < *P*_*g*_. The discussion is supported by the analysis of the *τ*(*P*) experimental data for glass-forming low molecular weight liquid *diisobutyl phthalate (DIIB, T*_*g*_*(0.1* *MPa)* = *196.8K)*, epoxy resin *bisphenol A/epichlorohydrin* (EPON 828, *T*_*g*_(0.1 *MPa*) = 253.9*K*) and liquid crystalline *isooctyloxycyanobiphenyl* (8*OCB, *T*_*g*_(0.1 *MPa*) = 220.7*K*). The latter vitrifies in the isotropic liquid phase, and the possible nematic phase is hidden below the glass transition. In given studies, pressures up to *P* ≈ 1.2 *GPa* were reached, i.e. for the domain hardly available in high resolutions tests carried out so far^8,10^. Experimental details are described in the Methods section.

## Results and Discussion

**Figure 1:**
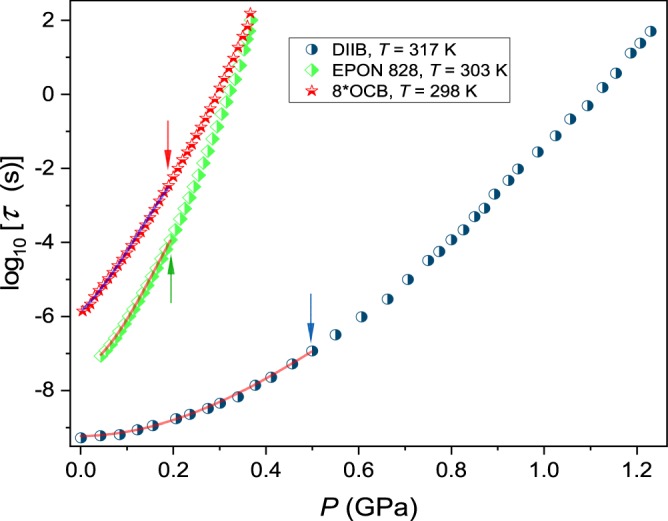
The isothermal pressure evolution of the structural relaxation time in previtreous domains of tested glass formers recalled in the Figure. Arrows show terminals of the reliable fitting using the simple approximation via Eq. (10a).

Figure 1 shows the pressure evolution of the structural relaxation time for selected isotherms for three qualitatively different glass formers 8*OCB, DIIP and EPON 828, in the pressure range 0.1 *MPa* < *P* < *P*_*g*_. They served for estimating both *V*^#^(*P*) and *V*_*a*_(*P*).

When discussing the physical meaning of *V*^#^(*P*) one can recall the definition of the pressure-related steepness index (the normalized rate of changes of the relaxation time, viscosity, …) in the previtreous domain^8^, which leads to the relation:

5 $${m}_{T}(P)=\frac{d\,{lo}{{g}}_{10}\tau (P)}{d\,(P/{P}_{g})}=\frac{{P}_{g}}{{ln}\,10}\frac{d\,{ln}\,\tau (P)}{dP},\,T=const$$

It terminates at the pressure-related fragility^11^: ${m}_{T}={m}_{T}({P}_{g})={[d{lo}{{g}}_{10}\tau (P)/d(P/{P}_{g})]}_{P={P}_{g}}$, which is the key metric for glass-forming ultraviscous/ultraslow systems. Then, the pressure-dependent (isothermic) coefficient *m*_*T*_(*P*) for *P* < *P*_*g*_ can be called the apparent fragility. Linking Eqs (3) and (5) one obtains the relation showing that *V*^#^(*P*) ∝ *m*_*T*_(*P*):

6 $${V}^{\#}(P)=(\frac{RT\,{ln}\,10}{{P}_{g}})\times {m}_{T}(P),\,T=const$$

Considering further the ratio of fragilities along *T*_*g*_(*P*) curve/line: $${m}_{P}(T)/{m}_{T}(P)=[d{lo}{{g}}_{10}\tau (T)/d({T}_{g}/T)]/$$$$[d{lo}{{g}}_{10}\tau (P)/d(P/{P}_{g})]=d(P/{P}_{g})/d({T}_{g}/T)$$ and linking this with Eq. (6) the following relations are obtained:

7a $${m}_{P}(T)={m}_{T}(P)\frac{T}{{P}_{g}}{(\frac{d{T}_{g}}{dP})}^{-1}$$

and

7b $${m}_{P}(T)=\frac{{V}^{\#}(P)}{R\,{ln}\,10}{(\frac{d{T}_{g}}{dP})}^{-1}$$

Equation (7b), originally derived in ref.^40^, is broadly used for calculating isobaric fragilities *m* = *m*_*P*_(*T*_*g*_) for different isobars, based on the knowledge of the ‘~activation volume *V*^#^(*P*)’ calculated via Eq. (3) and the pressure shift of *T*_*g*_^8,26–34,38–42,47–54^. Notwithstanding, Eq. (7a) is fundamentally more correct than Eq. (7b), since *V*^#^(*P*) should not be recalled as the apparent activation volume.

Recently, it was shown experimentally that changes of the pressure-related apparent fragility can exhibit a ‘universal’ previtreous behavior^5,7^:

8 $${m}_{T}(P)=\frac{A}{{P}^{\ast }-P}$$

where *T* = *const*, the amplitude *A* = *const*, and P^*^ is for the extrapolated singular pressure. Regarding pressures: *P* < *P*_*g*_ and P^*^ > *P*_*g*_.

**Figure 2:**
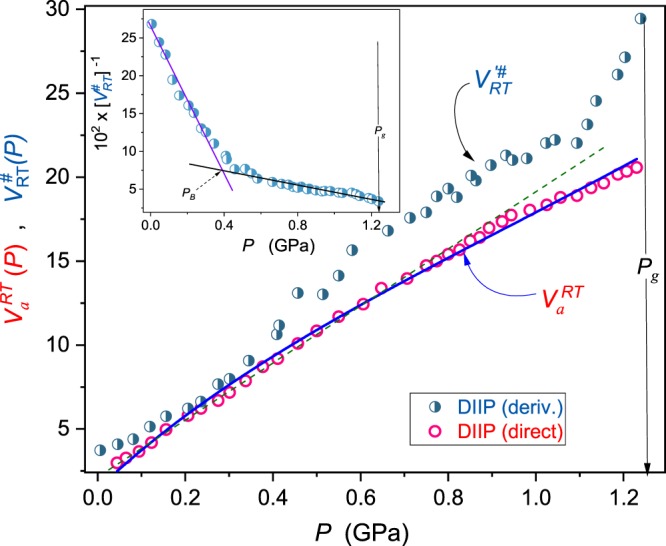
The pressure evolution of the normalized apparent activation volume *V*_*a*_(*P*)/*RT* (Eq. 9) and the reciprocal of *V*^#^(*P*)/*RT* ∝ *m*_*T*_(*P*) (Eqs 3 and 8: the inset) in superpressed diisobutylphtalate. In the inset, the manifestation of the dynamical crossover pressure *P*_*B*_ is indicated. Results are for *T* = 317 K isotherm. The thin dashed green line is for the Kornilov *et al*.^62^ moderate pressures approximation (Eq. (10b)). The thick blue curve is related to Eq. (13). The vertical arrow shows the glass transition. In the inset, the dynamic crossover pressure is indicated.

**Figure 3:**
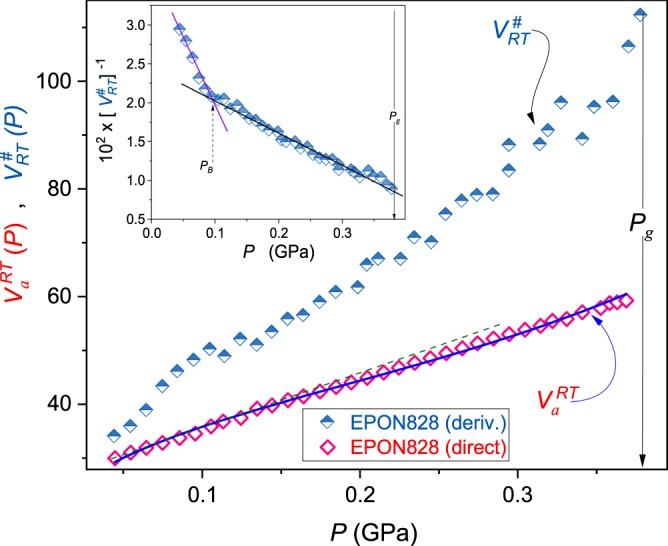
The pressure evolution of the normalized apparent activation volume *V*_*a*_(*P*)/*RT* (Eq. (9)) and the reciprocal of *V*^#^(*P*)/*RT* ∝ *m*_*T*_(*P*) (Eqs (3) and (8): the inset) in superpressed epoxy resin EPON 828. In the inset, the manifestation of the dynamical crossover pressure *P*_*B*_ is indicated. Results are for *T* = 303 K isotherm. The thin dashed green line is for the Kornilov *et al*.^62^ moderate pressures approximation (Eq. 10b). The thick blue curve is related to Eq. (13). The vertical arrow shows the glass transition. The dynamic crossover pressure is also indicated.

**Figure 4:**
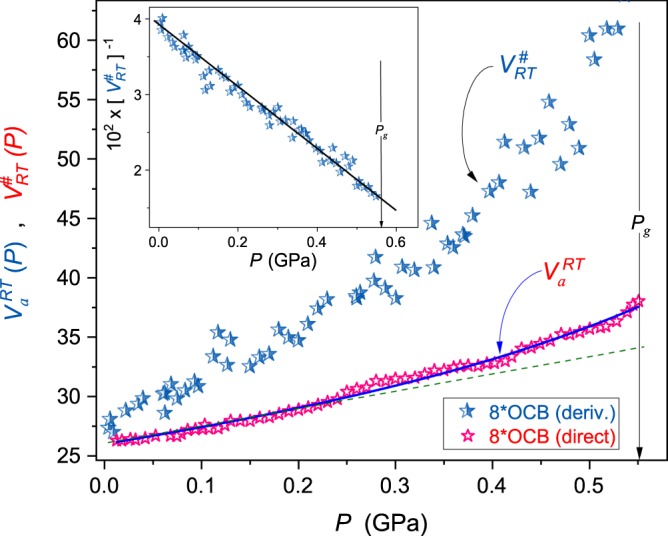
The pressure evolution of the normalized apparent activation volume *V*_*a*_(*P*)/*RT* (Eq. 9) and the reciprocal of *V*^#^(*P*)/*RT* ∝ *m*_*T*_(*P*) (Eqs (3) and (8): the inset) in superpressed liquid crystalline 8*OCB (isotropic liquid phase). Results are for *T* = 298 K isotherm. The thin dashed green line is for the Kornilov *et al*.^62^ moderate pressures approximation (Eq. (10b)). The thick blue curve is related to Eq. (13). The vertical arrow indicates the glass transition.

Following Eqs (6) and (8) one can conclude that: 1/*V*_#_(*P*) ∝ 1/*m*_*T*_(*P*) ∝ P^*^ − *P*. Such behavior is confirmed in the insets in Figs 2–4. One of the basic features of the previtreous domain is the appearance of two dynamical domains, i.e., regions with different SA or SB behavior remote and close to the glass transition, respectively^58,59^. This is associated with the crossover from the ergodic to the non-ergodic dynamical domain at (*T*_*B*_, *P*_*B*_), where *T*_*B*_ ≫ *T*_*g*_ and *P*_*B*_ ≪ *P*_*g*_^8,16,58,59^. Roland^60^ showed the pressure-temperature invariance of the dynamic crossover time-scale for a set of glass-forming liquids *τ*(*T*_*B*_, *P*_*B*_) ~ 10^−7±1^*s*. Until recently, the detection of *P*_*B*_ was associated with ${\Phi }_{P}(P)={(d{lo}{{g}}_{10}\tau (P)/dP)}^{-1/2}$ vs. *P* plot^8,59^, which is parallel to (*m*_*T*_(*P*))^−1/2^ vs. *P* presentation^5,7,56^. Such analysis assumes *a priori* the validity of the pressure counterpart of the Vogel-Fulcher-Tammann (VFT) relation for describing *τ*(*P*) experimental data^5,7,56^. Recently, an alternative and a model-free way for detecting the dynamic crossover via 1/*m*_*T*_(*P*) vs. *P* analysis was indicated^7^. This report shows that the pressure evolution of 1/*V*^#^(*P*) follows the pattern noted for 1/*m*_*T*_(*P*): this is shown in insets in Figs 2 and 3. The lack of *P*_*B*_ for 8*OCB in the inset in Fig. 4 results from the limited tested pressure range, between *P*_*g*_(*T*) = 0.56 *GPa* and *P* = 0.1 *MPa*. Consequently, in 8*OCB measurements were carried out only in the high-pressure dynamical domain (*P*_*g*_ > *P* > *P*_*B*_).

The above discussion shows the direct link between the magnitude considered so far as the apparent activation volume *V*^#^(*P*) and the apparent fragility *m*_*T*_(*P*). To determine the ‘real’ apparent activation volume *V*_*a*_(*P*) the protocol developed for the apparent activation energy *E*_*a*_(*T*) can be applied. The latter is given in refs^12–14^ and the Supplementary Info. For the pressure path, this means the numerical solution of the differential Eq. (4) for the given set of *τ*(*P*) experimental data. However, for the SB Eq. (1) a more straightforward way of determining *V*_*a*_(*P*) is possible since the prefactor in SB Eq. (1) is known: *τ*_0_^*P*^ = *τ*(*T*)_*P*__=__0_ ≈ *τ*(*T*)_*P*__=__0.1*MPa*_. Consequently, the apparent activation volume can be calculated directly from the SB Eq. (1), via the simple rearrangement:

9 $${V}_{a}(P)=\frac{RT}{P}\,{ln}(\frac{\tau (P)}{{\tau }_{0}^{P}})\,T=const$$

Results of such analysis present in main panels of Figs 2–4.

Insets in these Figures show pressure evolutions of 1/*V*^#^(*P*) ∝ 1/*m*_*T*_(*P*). It is visible that both quantities follow the same pattern, in agreement with Eq. (8) and ref.^7^. One can also see that *V*^#^(*P*) ~ *V*_*a*_(*P*) only for *P* → 0. Some distortion between *V*_*a*_(*P* → 0) and *V*^#^(*P* → 0) visible in Figs 2–4 can be associated with the distortions sensitive nature of Eq. (3) containing derivatives and used for calculating *V*^#^(*P*). For higher pressures, i.e. *P* → *P*_*g*_: *V*^#^(*P*) ≫ *V*_*a*_(*P*). Interestingly, the pressure evolution of the apparent activation volume *V*_*a*_(*P*) seems to be poorly, if at all, sensitive to the dynamical crossover phenomenon (see main parts of Figs 2 and 3).

To the best knowledge of the author, similar discussions of the apparent activation volume as introduced via Eq. (9) have appeared only in few reports so far. Beyeler and Lazarus^61^ considered the diffusion processes during chemical reactions under high compression and introduced a similar apparent activation volume concept. Recently, Kornilov *et al*.^62^ experimentally studied reaction rate constants for which the following relation was applied: $\mathrm{ln}\,k(P)$ = *a*′*P* + *b*′*P*^2^, for *P* < (220–300 *MPa*). It can be linked to the simple approximation of the SB Eq. (1):

10a $$\tau (P),\eta (P),k(P)\propto {\exp }\,(P\times \alpha (P))={\exp }(P\frac{a+bP}{RT})$$

Consequently, recalling Eq. (9) the activation volume is given via:

10b $${V}_{a}(P)=a+bP,\,T=const$$

Equations (10a) and (10b) describe *τ*(*P*) and *V*_*a*_(*P*) experimental data up 200 MPa for 8*OCB and EPON 828 and even above *P* ~ 400 MPa for DIIP, as shown by solid curves in Fig. 1 and thin (green) dashed lines in Figs 2–4.

When considering the description of *V*_*a*_(*P*) in the broad range of pressures one can recall a recently derived relation for the previtreous pressure evolution of *τ*(*P*), originating directly from ‘universal’ changes of the apparent fragility described via Eq. (8) ^7^:

11 $$\tau (P)={\tau }_{0P}{({P}^{\ast }-P)}^{-\Psi }$$

where the power exponent $\Psi =\,{ln}\,10(\varDelta {P}_{g}^{\ast }/{P}_{g}){m}_{T}({P}_{g})$, and the ‘discontinuity’ of the glass transition: Δ*P*_g_^*^ = P^*^ − *P*_*g*_^7^.

Combining Eqs (9) and (11) one obtains:

12 $${V}_{a}^{RT}(P)=\frac{{V}_{a}(P)}{RT}=\frac{C}{P}-\frac{\Psi }{P}\,{ln}({P}^{\ast }-P)\approx \frac{C-1}{P}+\frac{{({P}^{\ast })}^{-\psi }}{P}{(1-\frac{P}{{P}^{\ast }})}^{-\Psi }$$

where $$C={ln}\,{\tau }_{0P}/{ln}\,\tau (T)$$, *T* = *const*.

However, in Eq. (12) the apparent activation volume *V*_*a*_(*P*) → ∞ for *P* → 0, and then the anomalous increase occurs for *P* → 0.1 *MPa*. This problem can be avoided when taking into account that for solids, including liquids, the available range of pressures extends from the ‘normal’ positive domain (the isotropic compression) to the negative one, associated with the isotropic stretching^63^. This concept proved its fundamental significance for the general equation of state for water or critical mixtures and blends^63^. Angell and Quing experimentally showed the appearance of negative pressures and passing *P* = 0 without any hallmark, in glass-forming liquids using the centrifugal method^64^. Consequently, the ‘positive’ (isotropic compressing) and ‘negative’ (isotropic stretching) pressure regions can be considered as the common area, terminating at the absolute stability limit (SL) spinodal, hidden under negative pressures^63–65^. To describe experimental data in both pressure domains, one can consider the transformation *P* → Δ*P* = *P* − *P*_*SL*_^57^. Introducing the latter to Eq. (11) the modified dependence appears:

13 $${V}_{a}(P)=\frac{C}{\Delta P}-\frac{\Psi }{\Delta P}\,{ln}({P}^{\ast }-P)\approx \frac{C-1}{\Delta P}+\frac{{({P}^{\ast })}^{-\Psi }}{\Delta P}{(1-\frac{P}{{P}^{\ast }})}^{-\psi }$$

The above relation describes pressure changes of the apparent activation volume in the whole tested pressure range, up to *P* ≈ 1.2 *GPa*, as shown in Figs 2–4. It can be also extended into the negative pressures domain. Fitted parameters are given in Table 1. Values P^*^ were estimated from insets in Figs 2–4 using the condition 1/*V*^#^(P^*^) = 0; this also includes parameters *C* and Ψ estimated following ref.^7^.

**Table 1:** Results of fitting P^*^ via Eq. (13) for experimental data from Figs 2–4.

Glass former	P^*^ (GPa)	*C*	*ψ*	*P*_*SL*_ (GPa)
DIIB	2.6	72.3	74	−1.1
EPON 828	0.6	−12.1	27	−0.1
8*OCB	1.0	5.5	28.9	−0.21

Consequently, the analysis of *V*_*a*_(*P*) experimental data via Eq. (13) can be reduced solely to the single fitted parameter (*P*_*SL*_). This offers the new route for estimating the absolute stability limit pressure in negative pressures domain, which is considered as one of the most difficult to estimate properties via the experimental determination^63–65^.

## Conclusions

Concluding, the activation volume is the key parameter governing the complex previtreous dynamics for the structural relaxation time, viscosity, diffusion, and reaction rates under high pressure, as indicated in the SB Eqs (1) and (2). In numerous research reports, focusing on the glass transition, this property is considered to be given by ${V}^{\#}(P)=RT[d\,\mathrm{ln}\,\tau (P)/dP]$^8,26–35,38–42,47–55^, and subsequently used for developing the ‘pressure dimension’ of the glass transition models/theories^8,26–35,38–42,47–55^: the free volume model^66^, Adam-Gibbs model^67^, Cohen-Grest model^68^, and Avramov-Milchev model^69^. This report shows that the activation volume *V*_*a*_(*P*) ≠ *V*^#^(*P*). These magnitudes coincide only for the basic Barus dynamics, i.e. for Eq. (1) with *V*_*a*_ = *const*. The magnitude *V*^#^(*P*) is directly linked to the pressure-related apparent fragility *m*_*T*_(*P*).

The main result of the given report is the simple and non-biased way of determining the apparent activation volume *V*_*a*_(*P*) via Eq. (9) and the proposal for the parameterization of *V*_*a*_(*P*) evolution given by Eq. 13. It is worth mentioning that problems with the estimation and meaning of the activation volume seem to be absent for geophysics/deep Earth science where Murnaghan – O’Connel relation is broadly applied^70,71^:

14 $${V}_{a}(P)={V}_{a}^{ref.}{(1+\frac{P}{{K}_{0}/{K^{\prime} }_{0}})}^{1/{K^{\prime} }_{0}}$$

where *K*_0_, ${K^{\prime} }_{0}$ denotes 4/9 of the bulk modulus and its first derivative.

Notable is the similarity of Eq. (14) to approximated Eqs (12) and (13)], as shown above.

Results presented focused on the pressure evolution of the primary relaxation time, but they can also be applied for the viscosity, electric conductivity, diffusion, equilibrium, and reaction rates coefficients, in ultraviscous/ultraslow systems what indicates the broad range of fundamental and practical applications ranging from the glass transition physics and the solid state physics to ‘extreme’ chemistry, geophysics, petrology, innovative material engineering, high pressure preservation of food and biotechnology under pressure.

## Methods

In the last decades, the broadband dielectric spectroscopy (BDS) has become the key tool for studying previtreous behavior, including challenging insights from high-pressure studies^8,72,73^. In this report, BDS is used to determine the pressure evolution of the primary (*structural, alpha*) relaxation time^72,73^. Studies were carried out using the Novocontrol impedance analyzer, model 2015. BDS studies were carried out between the atmospheric pressure (*P* = 0.1 *MPa*) and the glass transition pressure, estimated via the empirical condition *τ*(*T*_*g*_, *P*_*g*_) = 100 *s*^8,72^. The structural relaxation times were determined from the peak frequencies of primary relaxation loss curves *ε"(f)*: *τ* = 1/2*πf*_*peak*_^8,9,72^. Tested samples were placed in the flat-parallel measurement capacitor made from Invar. The gap between plates *d* = 0.2 *mm* and their diameter 2*r* = 16 *mm*. Samples were entirely isolated from the pressurized medium (Plexol). They were in contact only with Invar, quartz (the spacer between plates) and Teflon. The pressure was transmitted to the sample via the deformation of 50 *mm* thick Teflon film. The process was supported by the computer–controlled pump, enabling pressure changes and programming with the precision Δ*P* = ±0.2 *MPa*, The pressure chamber was surrounded by a special jacket associated with the Julabo high-accuracy thermostat with the external circulation and the volume of the thermostated liquid *V* = 20*L*. These enabled temperature changes and control with accuracy Δ*T* = ±0.02 *K*. The temperature was monitored using the thermocouple within the pressure chamber and two platinum mini-resistors placed at the bottom and the top of the chamber. The high-pressure system was designed and produced by *UnipresEquipment* (Poland). Further experimental details are given in refs^6,7,74^. Notable, that the examined range of pressures was extended up to *P* ~ 1.2 *GPa*, the still hardly available range in high-resolution BDS pressure studies^8,26–54^. Experimental results cover time-scales from *τ*(*P* = 0.1 *MPa*) to *τ*(*P*_*g*_, *T*_*g*_) = 100*s*. The latter is commonly applied as the practical empirical estimation of (*T*_*g*_, *P*_*g*_)^8,10,26–54^.

## Supplementary information

The non-biased determining of the apparent activation energy in glass-forming system

## Data Availability

The data supporting the findings of this study are available from the author upon reasonable requests.

## References

[1] Kennedy D (2005). 125th Anniversary Issue: 125 outstanding problems in all of science: what is the nature of the glassy state. Science.

[2] Berthier L, Ediger M (2016). Facets of glass physics. Physics Today.

[3] Yoon H, McKenna. GB (2018). Testing the paradigm of an ideal glass transition: Dynamics of an ultrastable polymeric glass. Sci. Adv..

[4] Royall CP, Turci F, Tatsumi S, Russo J, Robinson J (2018). The race to the bottom: approaching the ideal glass?. J. Phys.: Condens. Matt..

[5] Drozd-Rzoska A (2019). Universal behavior of the apparent fragility in ultraslow glass forming systems. Sci. Rep..

[6] Rzoska SJ (2017). New challenges for the pressure evolution of the glass temperature. Front. Mater..

[7] Drozd-Rzoska A (2019). Pressure-related universal previtreous behavior of the structural relaxation time and apparent fragility. Front. Mater..

[8] Floudas, G., Paluch, M., Grzybowski, A. & Ngai, K. L. *Molecular Dynamics of Glass-Forming Systems. Effect of Pressure*. (Springer, Berlin, 2011).

[9] Donth, E. J. *The Glass Transition. Relaxation Dynamics in Liquids and Disordered Materials*. (Springer, Berlin, 2003).

[10] Rzoska, S. J., Mazur, V. & Drozd-Rzoska, A. *Metastable Systems under Pressure*. (Springer Verlag, Berlin, 2010).

[11] Hecksher T, Nielsen AI, Olsen NB, Dyre JC (2008). Little evidence for dynamic divergences in ultraviscous molecular liquids. Nat. Phys..

[12] Martinez-Garcia JC, Rzoska SJ, Drozd-Rzoska A, Martinez-Garcia JA (2013). Universal description of ultraslow glass dynamics. Nat. Comm..

[13] Martinez-Garcia JC, Rzoska SJ, Drozd-Rzoska A, Martinez-Garcia J, Mauro JC (2014). Divergent dynamics and the Kauzmann temperature in glass forming systems. Sci. Rep..

[14] Martinez-Garcia J, Rzoska SJ, Drozd-Rzoska A, Starzonek S, Mauro JC (2015). Fragility and basic process energies in vitrifying systems. Sci. Rep..

[15] Barus C (1893). Isothermals, isopiestic and isometrics relative to viscosity. Am. J. Sci..

[16] Starzonek S (2015). Fractional Debye–Stokes–Einstein behaviour in an ultraviscous nanocolloid: glycerol and silver nanoparticles. Soft. Matter.

[17] Whalley E (1964). Use of volumes of activation for determining reaction mechanisms. Adv. Phys. Org. Chem..

[18] Whalley E (1958). Conformational effects and the influence of pressure on reaction rates. Can. Chem..

[19] Williams G (1964). Complex dielectric constant of dipolar compounds as a function of temperature, pressure and frequency. Part 2. The a–relaxation of polymethyl acrylate. Trans. Faraday Soc..

[20] Arrhenius SA (1889). Über die reaktionsgeschwindigkeit bei der inversion von rohrzucker durch säuren. Z. Phys. Chem..

[21] Rzoska, S. J. & Mazur *Soft Matter under Exogenic Impacts*. (Springer, Berlin, 2008).

[22] van Eldik, R. & Jonas, J. *High Pressure Chemistry and Biochemistry*. (Springer, Berlin, 2011).

[23] Recio, M. J., Menendez, J. M. & de la Roca, A. O. *Introduction to High-Pressure Science and Technology*. (CRC Press., Boca Raton, 2015).

[24] Fornari, T. & Stateva, R. P., *High Pressure Fluid Technology and Green Food Processing*. (Springer, Berlin, 2014).

[25] Poirier, J. P. *Introduction to the Physics of the Earth’s Interior* (Cambridge Univ. Press., Cambridge, 2000).

[26] Casalini R, Paluch M, Fontanella J, Roland CM (2002). Investigation of the correlation between structural relaxation time and configurational entropy under high pressure in a chlorinated biphenyl. J. Chem. Phys..

[27] Paluch M, Casalini R, Best A, Patkowski A (2002). Volume effects on the molecular mobility close to glass transition in supercooled phenylphthalein-dimethylether. II. J. Chem. Phys..

[28] Pawlus S (2004). Temperature and volume effects on the change of dynamics in propylene carbonate. Phys. Rev. E.

[29] Kriegs H (2006). Pressure effects on the alpha and alpha’ relaxations in polymethylphenylsiloxane. J. Chem. Phys..

[30] Pawlus S, Mierzwa M, Paluch M, Rzoska SJ, Roland CM (2010). Dielectric and mechanical relaxation in isooctylcyanobiphenyl (8*OCB). J. Phys.: Condens. Matt..

[31] Grzybowski A, Koperwas K, wiety-Pospiech A, Grzybowska K, Paluch M (2013). Activation volume in the density scaling regime: Equation of state and its test by using experimental and simulation data. Phys. Rev. B.

[32] Paluch M (2014). General rules prospected for the liquid fragility in various material groups and different thermodynamic conditions. J. Chem. Phys..

[33] Panagos P, Floudas G (2015). Dynamics of poly(propyl methacrylate) as a function of temperature and pressure. J. Non-Cryst. Solids.

[34] Grzybowski AS, Urban S, Mróz S, Paluch M (2017). Activation volume of selected liquid crystals in the density scaling regime. Sci. Rep..

[35] Santamaría A, Boix M, Conde JI, Pascual B (2018). PVC/PBA random copolymers obtained by SET–DTLRP: Pressure effect on glass transition, rheology, and processing. J. Vinyl. Add. Technol..

[36] Hong L, Novikov VN, Sokolov AP (2011). Dynamic heterogeneities, boson peak, and activation volume in glass-forming liquids. Phys. Rev. E.

[37] Hong L, Gujrati PD, Novikov VN, Sokolov AP (2009). Molecular cooperativity in the dynamics of glass-forming systems: A new insight. J. Chem. Phys..

[38] Pawlus S, Paluch M, Nagaraj M, Vij JK (2011). Effect of high hydrostatic pressure on the dielectric relaxation in a non-crystallizable monohydroxy alcohol in its supercooled liquid and glassy states. J. Chem. Phys..

[39] Roland CM, Casalini R, Bergman R, Mattsson J (2008). Role of hydrogen bonds in the supercooled dynamics of glass-forming liquids at high pressures. Phys. Rev. B.

[40] Paluch M, Gapinski J, Patkowski A, Fischer EW (2001). Does fragility depend on pressure? A dynamic light scattering study of a fragile glass-former. J. Chem. Phys..

[41] Roland CM, Hensel-Bielowka S, Paluch M, Casalini R (2005). Supercooled dynamics of glass-forming liquids and polymers under hydrostatic pressure. Rep. Prog. Phys..

[42] Kamiński K (2012). The importance of the activation volume for the description of the molecular dynamics of glass-forming liquids. J. Phys.: Condens. Matt..

[43] Angell, C. A. Strong and fragile liquids. In, Ngai, K. L., and Wright, G. B. (eds) *Relaxations in Complex Systems*. (Natl. Tech. Inf. Serv., U.S. Dept. Comm., Springfield, 1985).

[44] Böhmer H, Ngai KL, Angell CA, Plazek DJ (1993). Nonexponential relaxations in strong and fragile glass formers. J. Chem. Phys..

[45] Ngai KL, Bao LA, Yee F, Soles CL (2001). Correlation of Positron Annihilation and Other Dynamic Properties in Small Molecule Glass-Forming Substances. Phys. Rev. Lett..

[46] Ho J, Govaert L, Utz M (2003). Plastic deformation of glassy polymers: correlation between shear activation volume and entanglement density. Macromolecules.

[47] Roland CM, Capaccioli S, Lucchesi M, Casalini R (2004). Adam-Gibbs model for the supercooled dynamics in the ortho-terphenyl ortho-phenylphenol mixture. J. Chem. Phys..

[48] Paluch M, Roland CM, Gapinski J, Patkowski A (2003). Pressure and temperature dependence of structural relaxation in diglycidylether of bisphenol A. J. Chem. Phys..

[49] Schwartz GA, Paluch M, Alegría Á, Colmenero J (2009). High pressure dynamics of polymer/plasticizer mixtures. J. Chem. Phys..

[50] Kamińska E, Tarnacka M, Jurkiewicz K, Kamiński K, Paluch M (2016). High pressure dielectric studies on the structural and orientational glass. J. Chem. Phys..

[51] Wojnarowska Z (2017). Experimental evidence of high pressure decoupling between charge transport and structural dynamics in a protic ionic glass-former. Sci. Rep..

[52] Wojnarowska Z (2017). How is charge transport different in ionic liquids? The effect of high pressure. Phys. Chem. Chem. Phys..

[53] Wojnarowska Z (2017). Effect of chain rigidity on the decoupling of ion motion from segmental relaxation in polymerized ionic liquids: Ambient and elevated pressure studies. Macromolecules.

[54] Urban S, Roland CM, Czub J, Skrzypek K (2007). Thermodynamic analysis of the low-frequency relaxation time in the Smectic A and C phases of a liquid crystal. J. Chem. Phys..

[55] White RP, Lipson JEG (2017). How free volume does influence the dynamics of glass forming liquids. ACS Macro Lett..

[56] Drozd-Rzoska A, Rzoska SJ (2006). On the derivative-based analysis for temperature and pressure evolution of dielectric relaxation times in vitrifying liquids. Phys. Rev. E.

[57] Drozd-Rzoska A, Rzoska SJ, Roland CM (2008). On the pressure evolution of dynamic properties in supercooled liquids. J. Phys.: Condens. Matt..

[58] Stickel F, Fisher EW, Richert R (1995). Dynamics of glass‐forming liquids. I. Temperature‐derivative analysis of dielectric relaxation data. J. Chem. Phys..

[59] Casalini R, Paluch M, Roland CM (2003). Dynamic crossover in supercooled liquids induced by high pressure. J. Chem. Phys..

[60] Roland CM (2008). Characteristic relaxation times and their invariance to thermodynamic conditions. Soft Matter.

[61] Beyeler M, Lazarus D (1971). Activation volume measurements. Z. Naturforsch..

[62] Kornilov DA, Kiselev VD, Konovalov AI (2017). Determination of the reaction acceleration effect at an elevated hydrostatic pressure. Russ. Chem. Bull. Int. Edit..

[63] Imre, A. R., Maris, H. J. & Williams, P. R. *Liquids under Negative Pressures*. (Kluwer, Dordrecht, 2002).

[64] Angell. CA, Qing Z (1989). Glass in a stretched state formed by negative pressure vitrification: trapping in and relaxing out. Phys. Rev. B.

[65] Pallares G (2014). Anomalies in bulk supercooled water at negative pressure. Proc. Natl. Acad. Sci. USA.

[66] Turnbull D, Cohen MH (1961). Free-volume model of the amorphous phase: glass transition. J. Chem. Phys..

[67] Adam G, Gibbs JH (1965). On the temperature dependence of cooperative relaxation properties in glass-forming liquids. J. Chem. Phys..

[68] Cohen MH, Grest GS (1977). Liquid-glass transition, a free-volume approach. Phys. Rev. B..

[69] Avramov I, Milchev A (1998). Effect of disorder on diffusion and viscosity in condensed systems. J. Non-Cryst. Solids.

[70] Murnaghan FD (1944). The compressibility of media under extreme pressures. Proc. Natl. Acad. Sci. USA.

[71] O’Connell RJ (1977). On the scale of mantle convection. Tectonophysics.

[72] Kremer, F. & Schönhals, A. (eds) *Broadband Dielectric Spectroscopy*. (Springer, Berlin, 2013).

[73] Kremer, F. & Loidl, A. *Scaling of Relaxation Processes*. (Springer, Berlin, 2018).

[74] Rzoska, S. J., Drozd-Rzoska, A. & Starzonek, S. Nonlinear dielectric effect in critical liquids, pp. 1–35: in Richert, R. (ed.) *Nonlinear Dielectric Spectroscopy: Springer Series Advanced Dielectrics*. (Springer, Berlin, 2018).

