# Supplementary material for: Activation volume in superpressed glass-formers
Source: Sci Rep. 2019 Sep 24;9:13787. doi: 10.1038/s41598-019-49848-w (PMC6760521; doi:10.1038/s41598-019-49848-w)
Supplement: Supplementary file 1 — The non-biased determining of the apparent activation energy in glass-forming system [file 41598_2019_49848_MOESM1_ESM.pdf]

Activation volume in superpressed glass-formers

Aleksandra Drozd-Rzoska

Institute of High Pressure Physics Polish Academy of Sciences,
ul. Sokołowska 29/37.01-142 Warsaw, Poland

SUPPLEMENTARY INFORMATION:

The non-biased determining of the apparent activation energy in glass-forming systems

The temperature evolution of viscosity or the structural relaxation time in glass-forming systems under atmospheric pressure is described by the Super-Arrhenius (SA) relation with the apparent (changeable) activation energy $E_a(T)$:[S1]

$$\tau(T) = \tau_0 \, exp\left(\frac{E_a(T)}{RT}\right) \qquad \eta(T) = \eta_0 \, exp\left(\frac{E_a(T)}{RT}\right) \qquad \text{(s1)}$$

Often the 'quasi-universal' value of the prefactor $\tau_0 = 10^{-14} s$ is assumed,[S1] what is subsequently used for calculating changes of the apparent activation energy on approaching the glass temperature T_g via:[S2]

$$E_a = RT \, ln\left(\frac{\tau(T)}{\tau_0}\right) \qquad \text{(s2)}$$

The glass temperature is empirically associated with the time-scale $\tau(T_g, P_g) = 100 s$ or viscosity $\eta(T_g, P_g) = 10^{13} Poise$.[S1,S2] Notwithstanding, Eq. (s2) yields qualitatively biased values of the apparent activation volume, since for different glass formers $10^{-11} < \tau_0 < 10^{-16}$ and cannot be exactly known in prior.[S3] The non-biased way of estimating the apparent activation energy was proposed in ref.[S3] basing on the numerical solution, for given sets of $\tau(T)$ or $\eta(T)$ experimental data, of the differential equation directly resulted from Eq. (s1):[S3]

$$R\frac{d\,ln\tau(T)}{d(1/T)} = E_a(T) + \frac{dE_a(T)}{d(1/T)} \quad \text{(s3)}$$

In refs.[S3,S4,S5] this way of calculating $E_a(T)$ was used for obtaining the apparent activation energy temperature index $I_{DO}(T) = -dl\,ln\tau(T)/dT$ for which the 'universal' previtreous behavior was found $I_{DO}(T) = nT_N/(T - T_N)$, where $T_N < T_g$ is the extrapolated singular temperature and the parameter $0.18 < n < 1.52$. The value $n = 1$ is coupled to the Vogel-Fulcher-Tammann equation[S1] for $\tau(T)$ or $\eta(T)$ portrayal and no-symmetry systems.[S3]

Supplemental References:

S1. Donth, E. J. *The Glass Transition. Relaxation Dynamics in Liquids and Disordered Materials* (Springer, Berlin, 2003).

S2. Hecksher, T., Nielsen, A. I., Olsen, N. B. & Dyre, J. C. Little evidence for dynamic divergences in ultraviscous molecular liquids. *Nat. Phys*. **4**, 737-741 (2008).

S3. Martinez-Garcia, J., C., Rzoska, S. J., Drozd-Rzoska A., and Martinez-Garcia, J. A. Universal description of ultraslow glass dynamics. *Nat. Comm.* **4**, 1823 (2013).

S4. Martinez-Garcia, J. C., Rzoska, S. J., Drozd-Rzoska, A., Martinez-Garcia, J., and Mauro, J. C. Divergent dynamics and the Kauzmann temperature in glass forming systems. *Sci. Rep.* **4**, 5160 (2014).

S5. Martinez-Garcia, J., Rzoska, S. J., Drozd-Rzoska, A., Starzonek, S., and Mauro, J. C. Fragility and basic process energies in vitrifying systems. *Sci. Rep.* **5**, 8314 (2015).
